# The miR-28-5p Targetome Discovery Identified SREBF2 as One of the Mediators of the miR-28-5p Tumor Suppressor Activity in Prostate Cancer Cells

**DOI:** 10.3390/cells9020354

**Published:** 2020-02-03

**Authors:** Sofia Fazio, Gabriele Berti, Francesco Russo, Monica Evangelista, Romina D’Aurizio, Alberto Mercatanti, Marco Pellegrini, Milena Rizzo

**Affiliations:** 1 Non-coding RNA Laboratory, Institute of Clinical Physiology (IFC), CNR, 56124 Pisa, Italy; sofia.fazio@univ-cotedazur.fr (S.F.); gabriele.berti@hotmail.it (G.B.); m.evangelista@ifc.cnr.it (M.E.); alberto.mercatanti@ifc.cnr.it (A.M.); 2 Centre Méditerranéen de Médecin Moléculaire INSERM U1065, Université Côte d’Azur, 06204 Nice, France; 3 Institute of Informatics and Telematics (IIT), CNR, 56124 Pisa, Italy; francesco.russo@cpr.ku.dk (F.R.); romina.daurizio@gmail.com (R.D.); marco.pellegrini@iit.cnr.it (M.P.); 4 Novo Nordisk Foundation Center for Protein Research, Faculty of Health and Medical Sciences, University of Copenhagen, 2200 Copenhagen, Denmark; 5 Tuscan Tumor Institute (ITT), 50139 Firenze, Italy

**Keywords:** prostate cancer, miRNA targetome, SREBF2, miR-28-5p, miRNA pull out assay, microRNA

## Abstract

miR-28-5p is downregulated in some tumor tissues in which it has been demonstrated to have tumor suppressor (TS) activity. Here, we demonstrate that miR-28-5p acts as a TS in prostate cancer (PCa) cells affecting cell proliferation/survival, as well as migration and invasion. Using the miRNA pull out assay and next generation sequencing, we collected the complete repertoire of miR-28-5p targets, obtaining a data set (miR-28-5p targetome) of 191 mRNAs. Filtering the targetome with TargetScan 7, PITA and RNA22, we found that 61% of the transcripts had miR-28-5p binding sites. To assign a functional value to the captured transcripts, we grouped the miR-28-5p targets into gene families with annotated function and showed that six transcripts belong to the transcription factor category. Among them we selected SREBF2, a gene with an important role in PCa. We validated miR-28-5p/SREBF2 interaction, demonstrating that SREBF2 inhibition affects almost all the tumor processes altered by miR-28-5p re-expression, suggesting that SREBF2 is an important mediator of miR-28-5p TS activity. Our findings support the identification of the targetome of cancer-related miRNAs as a tool to discover genes and pathways fundamental for tumor development, and potential new targets for anti-tumor therapy.

## 1. Introduction

miR-28-5p is a miRNA with a tumor suppressor (TS) activity downregulated in several tumor tissues [1,2,3,4,5,6]. Increasing evidences indicate that miR-28-5p inhibited some well-characterized oncogenes such as CCND1, HOXB3 [4] in colorectal carcinoma and IL-34 [7] and IGF-1 [1] in hepatocellular carcinoma.

It has been demonstrated in vitro that miR-28-5p exerts its tumor suppressor activity by affecting several aspects of tumor cell biology. miR-28-5p re-expression inhibited cell proliferation in B-cell lymphomas and renal cell carcinoma regulating BAG1 [3] and RAP1B [2] expression, respectively. Moreover, miR-28-5p inhibited cell migration and invasion in gastric cancer by repressing the AKT phosphorylation levels [6]. Interestingly, miR-28-5p, by regulating the triose-phosphate isomerase (TPI) in colorectal carcinoma, contributed to the increased glycolytic capacity of the tumor cells [8]. Recently, two reports indicate that the capability of miR-28-5p to inhibit its targets is also regulated by the sponge effect of long non-coding RNAs, such as UCA1 [5] and CCAT1 [9]. Although some reports about the molecular targets through which the miR-28-5p exerts its TS activity in different tumor types are available, most experimental studies validate only one or a few miRNA targets at once. However, a single miRNA simultaneously interacts and regulates hundreds of mRNA molecules, generating a complex regulatory network.

Previously, we demonstrated, for the first time, that miR-28-5p is downregulated in two prostate cancer (PCa) cell lines, and that it acts as a TS in PCa cells [10]. Here we explored the complete repertoire of the miR-28-5p targets in a PCa cell line. We chose to use an experimental rather than a computational approach to reduce false positives. Several high-throughput experimental methods are now available, most of which are based on the sequencing (NGS) of RNA isolated by the crosslinking and immunoprecipitation of Argonaute (AGO), such as HITS-CLIP [11], PAR-CLIP [12], iPAR-CLIP [13] and CLASH [14]. Since these techniques allow the isolation of the RNA molecules that interact with all miRNAs, the major disadvantage is that they favor the identification of the targets of the highly expressed miRNAs. To overcome this limitation, we performed an miRNA pull out assay [15] that is based on the capture of the specific miRNA/target complexes through the transfection of a biotinylated version of the miRNA of interest. We already used this technique associated with the microarray for target identifications [16], and more recently we combined the miRNA pull out assay with NGS technology to identify the miR-26a-5p targetome in PCa cells [17]. 

In the present work we isolated and identified the mRNA targets associated with miR-28-5p (miR-28-5p targetome) in a PCa cell line. We focused on SREBF2, validating miR-28-5p/SREBF2 interaction and demonstrating that SREBF2 inhibition exerts TS activity in PCa cells.

## 2. Materials and Methods

### 2.1. Cells and Culture Conditions

DU-145, 22Rv1 and LNCaP cells were grown in RPMI Medium 1640 (EuroClone) whereas PC3 cells were grown in HAM’s Medium (Euroclone, Milan, Italy), V-CaP cells in DMEM (EuroClone) and HCT116 Dicer^-/-^ cells in McCOY’s (EuroClone). All the media were completed by adding 10% FBS (Fetal Bovine Serum, EuroClone), 1% penicillin/streptomycin (EuroClone) and 1% L-glutamine (Sigma-Aldrich). The cells were incubated at 37 °C in a humidified atmosphere containing 5% CO_2_.

### 2.2. Transfection

Transient transfections of miRNAs mimics (miR-28a-5p), miRNA inhibitor (d-28-5p), SREBF2 inhibitor (siR-SREBF2) or control (CT) (GenePharma, Shanghai, China) in DU-145, LNCaP or HCT116 Dicer^−/−^ cells, were performed using Lipofectamine 2000 (Thermo Fisher, Waltham, MA, USA) following the manufacturer’s protocol. Briefly, 1.5 × 10^5^ cells were seeded in P30 dishes and transfected after 48 h using 10 µL of Lipofectamine. The transfected cell suspension was used for cellular and molecular assays.

### 2.3. Cell Proliferation

1 × 10^5^ DU-145 or LNCaP cells were seeded in a series of P30 dishes and transfected with either miR-28-5p mimic/inhibitor, SREBF2 inhibitor, or control. 48 h after transfection, these cells were collected and counted using the Bürker chamber.

### 2.4. Survival Assay

To evaluate cell survival, cells were seeded at a cell density of 200 cells/P60 dishes to allow colony formation. Dishes were stained after 10–12 days with 0.1% Crystal Violet in 20% methanol, and the fraction of surviving cells was calculated as the ratio between number of colonies/number of seeded cells.

### 2.5. Soft Agar Colony Formation Assay

1 × 10^5^ DU-145 cells were resuspended in culture medium plus 0.3% agarose and plated on an agarose base (RPMI plus 0.6% agarose, prepared the day before) in a well of a 6-well plate. After 10–15 days, the colonies that were visible in five randomly chosen microscope fields were counted using a 10× objective.

### 2.6. Wound Healing Assay

2 × 10^5^ DU-145 cells were seeded in the two chambers of a culture insert (IBIDI, Martinsried, Germany) previously placed on a P30 dish. The insert was removed at 80% of cell confluence (0 h) in order to leave the cells free to migrate and to fill the empty space. The cells were observed at 6, 9 and 13 h after the insert removal, with 10× and 20× objectives. The images were taken using a Leica DM IL light emitting diode (LED) microscope and analyzed with ImageJ. The migratory rate of each sample was calculated as the relative percentage of gap closure at 13 h versus 0 h.

### 2.7. Transwell Assay

The 24-well cell culture chamber (Falcon) with 0.4 μm PET membrane covered (invasion assay) or not (migration assay) with 300 μg/mL Matrigel (BD) was used to measure the migratory or invasive rate of DU-145 in different conditions. 5 × 10^4^ cells were inoculated onto the upper chamber in a serum free medium and the medium with serum and growth factor were added in the lower chamber and incubated for 13 h. The non-invading/migrated cells on the upper surface of the upper chamber were wiped off with cotton swab, whereas the invading/migrated cells on the lower surface of the upper chamber were fixed with 2% paraformaldehyde and stained with 0.1% crystal violet dissolved in 20% methanol and allowed to be dried at room temperature. Lysis was performed with 10% acetic acid and the optical density (OD 590 nm) of the solution was detected with a ChroMate microplate reader (Awareness Technology, Westport, CT, USA), and used to measure cell proliferation.

### 2.8. miRNA Pull Out Assay

The miRNA pull out assay was performed as described in Rizzo et al. [17]. DU-145 was transfected using Lipofectamine 2000 (Thermo Fisher, Waltham, MA, USA) with 60 nM of either miR-28-5p duplex (ds-miR-28_CT_) or a mix of 3′ biotin-tagged miR-28-5p 8tU (nucleotide 8 was a thiouridine) and miR-28-5p 18tU duplexes (ds-miR-28_BIO_) (Supplementary Figure S1). All oligos were synthetized by Bio-Synthesis Inc. (Lewisville, TX, USA), and 24 h after transfection, these cells were irradiated with ultraviolet (UV) (365 nm, 2J/cm^2^) using the Bio-Link crosslinking (BLX) (Vilmer Lourmat, Marne-la-Vallée, France) and total RNA extracted with TRIzol (Thermo Fisher, Waltham, MA, USA) directly on adherent cells following the manufacturer’s protocol (Supplementary Figure S2). 15 μg of RNA was incubated 4 h at 4 °C with 100 μL of streptavidin-conjugated beads (Streptavidin Sepharose high performance, GE Healthcare, Chicago, IL, USA) and the RNA complexed with the beads was recovered using TRIzol. We performed two biological replicates obtaining two miR-28_CT_ (control) and two miR-28_BIO_ (miR-28-5p) pull out samples consisting of background RNA and ds-miR-28_BIO_/interacting mRNA complexes, respectively.

### 2.9. Targets Identification by Next Generation Sequencings (NGS)

The RNA obtained after the miRNA pullout procedure (Supplementary Figure S2) from both miR-28_CT_ (background RNA) and miR-28_BIO_ pull out samples was used for the construction of the cDNA libraries using the TruSeq Stranded Total RNA Sample Preparation kit (Illumina, San Diego, CA, USA) according to the manufacturer’s suggestions. cDNA libraries were sequenced using the HiSeq2000 (Illumina) in single-reads mode (50bp) obtaining about 20 million reads for each sample (fastq files).

### 2.10. NGS Data Analysis

NGS data (GEO accession number GSE143589) were analyzed using the Galaxy platform (https://usegalaxy.org/), by applying several tools: FASTQC to check the quality of the raw sequence data, TopHat2 for the alignment of the reads on human genome (hg38) and Cufflinks set of tools to estimate the abundance of transcripts. Specifically, Cuffdiff was used to perform differential expression analysis, and we found transcripts enriched in the miR-28_BIO_ in comparison to miR-28_CT_ pull out samples. We selected the enriched transcripts with q-value < 0.05 (miR-28-5p targetome) (Supplementary Figure S2).

### 2.11. Bioinformatics Analyses

The available miRNAs-mRNAs expression data and clinical parameters of patients of MSKCC Prostate Oncogenome Project [18] were retrieved from cBioPortal (http://www.cbioportal.org/; GEO accession number: GSE21032). This dataset consists of 99 primary and 14 metastatic tumor tissues and 27 normal controls. Nonparametric tests (i.e., Wilcoxon, Kruskal–Wallis and Spearman rank-correlation) and recurrence-free survival analysis were conducted in R. In more details, the probabilities of biochemical recurrence (BCR) events, defined as PSA ≥ 0.2 ng/mL on two occasions, were estimated with the Kaplan–Meier method (Kaplan–Meier, 1958) and then compared between groups with log-rank statistics using “survival” R packages.

For the correlation analysis between miR-28-5p expression level and Gleason score/Tumor T stage, The Cancer Genome Atlas (TCGA) Prostate Adenocarcinoma (PRAD) primary tumors (*n* = 494) data were investigated from work by the Broad Institute TCGA Genome Data Analysis Center (2016) [19].

### 2.12. Bioinformatics Analyses Related to miRNA Pull Out Assay

To identify the miR-28-5p predicted targets in the miR-28-5p targetome, we performed a target prediction analysis by using the script version of TargetScan 7 [20], PITA [21] and RNA22 [22] (Supplementary Figure S2). The different algorithms have different settings and filters. For PITA and RNA22 we applied the filter for a maximum of one mismatch and one G:U in the seed match. Moreover, for PITA we selected a score (i.e., the ddG score based on the folding energy) ≤ −10. For RNA22 thresholds for the folding energy ≤ −10 and a *p*-value ≤ 0.05 were applied. We considered putative targets if at least one target prediction algorithm was able to detect the interaction. The 3′UTR sequences of the transcripts belonging to the miR-28-5p targetome were obtained by using UCSC Table Browser (http://genome.ucsc.edu/).

For the Gene Set Enrichment Analysis, the GSEA online tool (http://software.broadinstitute.org/gsea/msigdb/index.jsp) was used to discover enriched gene functions (q < 0.05), setting the MSigDB (the Molecular Signatures Database) as annotated gene lists.

To evaluate the presence of putative miR-28-5p binding sites in the SREBF2 ORF and 5′UTR we used RNAhybrid [23] considering the binding sites with the MFE (minimum free energy) value ≤ –24 Kcal/mol. We also confirmed the predicted binding site using PITA and RNA22.

### 2.13. Quantification of miRNAs and mRNAs (qRT-PCR)

Total RNA was extracted from 1 × 10^6^ cells using the miRNeasy mini kit (Qiagen, Hilden, Germany) following the manufacturer’s protocol. 1 µg of total RNA was used for the reverse transcriptase reaction, performed, according to the manufacturer’s recommendation, using either the QuantiTect reverse Transcription kit (Qiagen) or the miScript II RT kit (Qiagen) to retrotranscribe mRNAs or mature miRNAs, respectively. The quantification of mRNAs and mature miRNAs were performed with Rotor-Gene Q 2plex (Qiagen), using the SsoAdvanced™ SYBR^®^ Green Supermix (BIO-RAD, Hercules, CA, USA) or the miScript SYBR Green PCR Kit (Qiagen) respectively, according to the manufacturer’s protocol. The relative quantification was performed using the Rotor-Gene Q Software, by normalizing to the internal controls (GAPDH, ACTB and HPRT for mRNAs and U6, SNORD55 and SNORD110 for mature miRNAs). The miR-28a-5p relative expression level in tumor cell lines was determined with respect to RNA from normal cells (FirstChoice human total RNA, Ambion, Austin, TX, USA). All reactions were performed in triplicate, and the results are expressed as the mean of at least three biological replicates.

### 2.14. Western Blot Analysis

Proteins were extracted from cell pellets using Lysis Buffer (1 M Tris HCl pH 8, Triton × 100 1%, Na deoxycholate 0.25%) with the addition of PMSF 1mM. Proteins quantification was performed with the colorimetric method using the Biorad protein Assay Reagent (BIO-RAD). The ChroMate microplate reader (Awareness Technology, Westport, CT, USA) was used to measure the absorbance at 595 nm. To perform Western blot, proteins were separated on polyacrylamide gels SDS-PAGE (10%, gel precast MINI-PROTEAN^®^ TGX Stain-Free^TM^, BIO-RAD), and transferred to 0.2 μm nitrocellulose membranes by electro blotting using the Trans-Blot Turbo Blotting System (BIO-RAD). The membranes were then blocked with 5% nonfat dry milk solution in TBST. Anti GAPDH (Cell Signaling) (1:20,000) and anti SREBF2 (Santa Cruz Biotechnology, Dallas, TX, USA) (1:500) primary antibodies were used to incubate the membranes overnight at 4 °C followed by a 1-h incubation with the recommended secondary antibody. Bands were revealed using ECL (GE Healthcare, Chicago, IL, USA). Images were detected and protein quantified using the ChemiDoc system (BIO-RAD).

### 2.15. Vectors Construction

The sequence of the 3′UTR (ENSG00000198911: 31-1763, pSREBF2_3’UTR_), the 5′UTR and first part of the ORF (ENSG00000198911: 1753-3611, pSREBF2_5’UTR-ORFI_) and the second part of the ORF (ENSG00000198911: 3696-5213, pSREBF2_ORFII_) were cloned downstream of the luciferase coding sequence into the pmiR vector (pMIR-Report Luciferase, Ambion). The miR-28-5p sensor was built by cloning two perfect match sequences complementary to miR-28-5p sequence downstream from the luciferase in the pmiR vector.

### 2.16. Luciferase Reporter Assay

To investigate the interaction between the miR-28-5p and SREBF2 transcript, 1.5 × 10^5^ HCT116 Dicer^−/−^ colon cancer cells were seeded in each well of a 12-well plate and after 24 h they were co-transfected with 150 ng of pSREBF2_3’UTR_ (or pSREBF2_5’UTR-ORFI_ or pSREBF2_ORFII_), 100 ng of pRL control vector-TK (Renilla, Promega) and with 60 nM of miR-28-5p mimic (or CT). The transfection reaction was performed using Lipofectamine 2000 (Thermo Fisher) according to the manufacturer’s instructions. The luciferase assay was performed one day after transfection, using the Dual-Luciferase Reporter Assay System (Promega, Madison, WI, USA), according to the manufacturer’s instructions. The luminometer GloMax-Multi Detection System (Promega,) was used to evaluate the luminescence, and the pRL-TK (Renilla) was used to normalize the luminescence arising from pmiR-SREBF2 vectors.

### 2.17. Statistical Analyses

All experimental results are expressed as mean SD of at least three independent experiments. Data were analyzed with Student’s *t*-test (* *p* < 0.05, ** *p* < 0.01, *** *p* < 0.001).

## 3. Results

### 3.1. miR-28-5p Showed Antitumor Effects in PCa

We previously demonstrated that miR-28-5p is downregulated in the androgen independent PC-3 and DU-145 PCa cell lines, and that its re-expression in DU-145 cells exerts a tumor suppressor activity by reducing cell proliferation/survival, increasing apoptosis and inducing an increase of cells in G1 phase [10].

**Figure 1:**
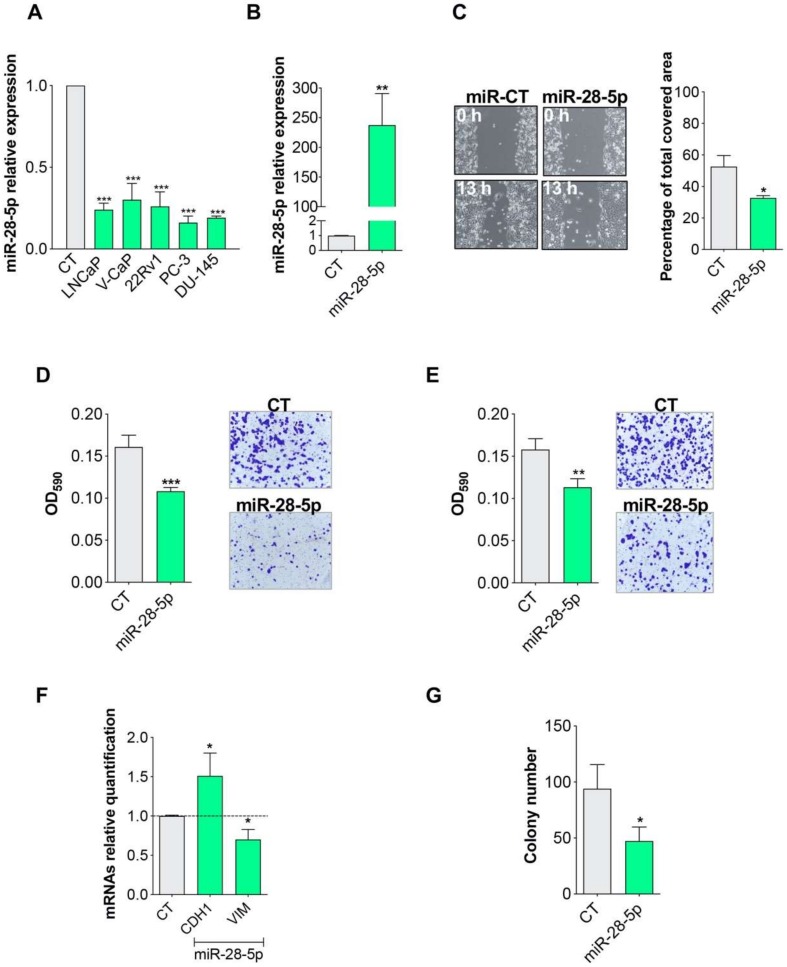
Effect of miR-28-5p re-expression in PCa cells. (**A**) Analysis of the miR-28-5p expression level by qRT-PCR in prostate cancer cell lines with respect to the normal cells RNA. (**B**) Relative expression level of miR-28-5p, evaluated by qRT-PCR, after miR-28-5p transfection in DU-145 cells. Cell migration (**C**,**D**) and invasion (**E**) of DU-145 cells after miR-28-5p overexpression evaluated by wound healing assay (**C**) and trans-well assay (**D**,**E**). (**F**) Relative expression of E-cadherin 1 (CDH1) and vimentin (VIM) in miR-28-5p overexpressing versus normal DU-145 cells. (**G**) Number of colonies formed in soft agar in DU-145 cells after miR-28-5p or CT overexpression. * *p* < 0.05, ** *p* < 0.01, *** *p* < 0.001, unpaired *t*-test.

In this paper, we first measured miR-28-5p level in a larger number of PCa cell lines, demonstrating that this miRNA was generally downregulated in PCa in vitro (Figure 1A). To investigate whether miR-28-5p re-expression plays a role in PCa cell migration and invasion, we overexpressed miR-28-5p (Figure 1B) in DU-145 cells and performed both a wound healing assay (Figure 1C) and trans-well assays (Figure 1D,E). The results showed that miR-28-5p is able to inhibit both the migration (Figure 1C,D) and the invasion (Figure 1E) ability of DU-145 cells. In line with these results, the expression of the epithelial marker E-cadherin 1 (CDH1) and the mesenchymal marker vimentin (VIM) increase and decrease, respectively, after miR-28-5p overexpression (Figure 1F). We also evaluated the anchorage-independent growth using the soft agar colony formation assay after miR-28-5p re-expression. The number of anchorage-independent colonies was significantly decreased after miR-28-5p re-expression (Figure 1G). These data support the tumor suppressor role of miR-28-5p by acting in various aspects of tumor biology.

**Figure 2:**
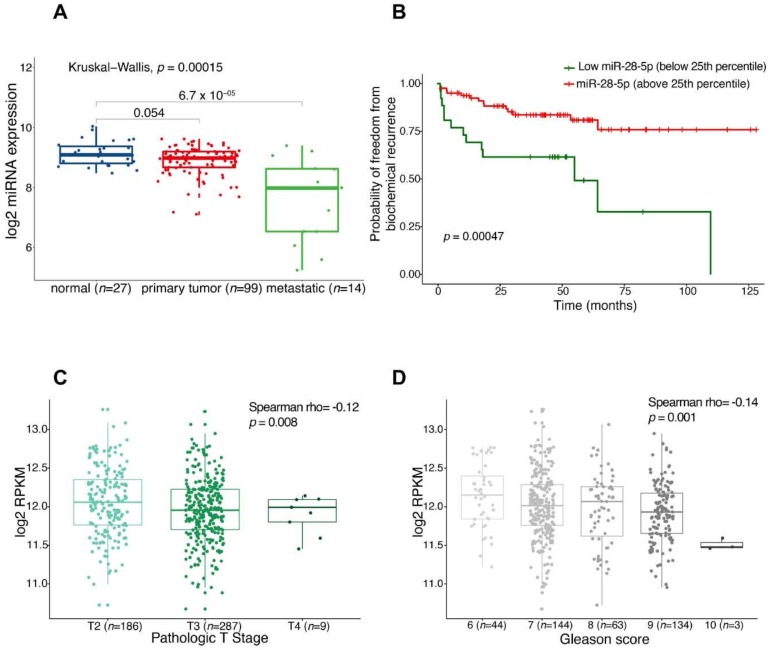
Association of miR-28-5p expression with clinical parameters in PCa patients. (**A**) Analysis of miR-28-5p expression level data of the Memorial Sloan Kettering Cancer Center (MSKCC) study’s patients. The significances according to the Kruskal–Wallis and Wilcoxon test are indicated. (**B**) Kaplan–Meier curves for recurrence-free survival events between MSKCC patients after dividing samples into two groups according to the 25st quartile of miR-28-5p expression level. Log-rank test’s *p*-value is shown. The miR-28-5p expression levels (log2RPMK) of TGCA PRAD samples are shown and grouped by pathological T stage (**C**) and Gleason score (**D**) with *p*-value from the Spearman’s test of association.

To evaluate the relevance of miR-28-5p in PCa tumorigenesis we first checked the expression of this miRNA in tumor/normal tissues of PCa patients using the miRNAs expression data deposited in cBioPortal database. We verified that miR-28-5p tends to be less expressed in primary tumors and shows a significant downregulation in metastatic tissues (Figure 2A). These results are in line with the anti-invasive/-migratory ability of miR-28-5p re-expression.

In addition, we found that patients with lower miR-28-5p or LPP expression (the miR-28-5p host gene, that display an expression pattern resembling the one of miR-28-5p, Supplementary Figure S3A,B) presented a significantly lower recurrence-free survival (Figure 2B for miR-28-5p and Supplementary Figure S3C for LPP). Finally, exploiting the primary tumor samples from TCGA Prostate Adenocarcinoma Study (n = 494), we found that miR-28-5p expression is inversely associated with both the pathological T stage (Spearman rho = −0.12, *p*-value = 0.008, Figure 2C) and the Gleason score (Spearman rho = −0.14, *p*-value = 0.001, Figure 2D).

Overall data demonstrated that miR-28-5p acts as a TS-miRNA in PCa cells regulating key pathways involved not only in tumor cell proliferation and survival, but also invasion and migration, and suggested that this miRNA plays a role in tumor progression in vivo.

### 3.2. Identification of miR-28-5p/Targetome

**Figure 3:**
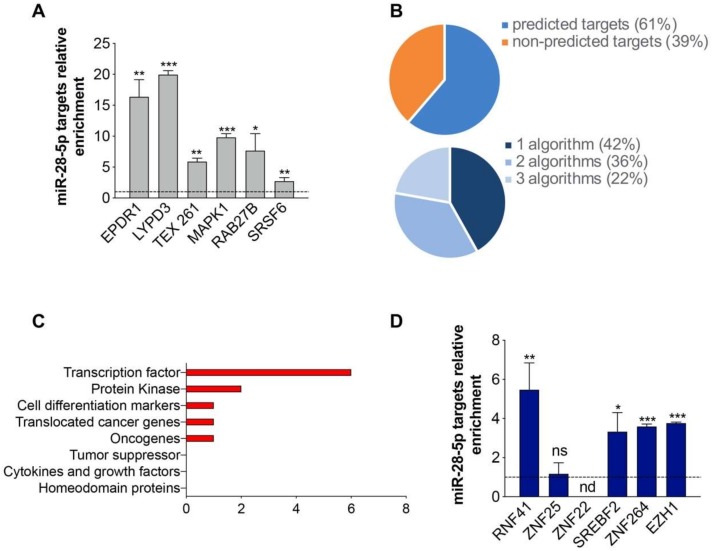
Analysis of miR-28-5p targetome. (**A**) Evaluation of the enrichment of miR-28-5p selected targets in the miR-28_BIO_ compared to miR-28_CT_ pull out samples by qRT-PCR. (**B**) Pie chart representing the percentage of the predicted/non-predicted targets (upper panel) and the percentage of the targets predicted by one, two or three algorithms (lower panel) in the miR-28-5p targetome. (**C**) Results of the MSigDB gene sets analyses showing the identified genes families. (**D**) Enrichment of the miR-28-5p targets belonging to the “transcription factors” family in the miR-28_BIO_ compared to miR-28_CT_ pull out samples by qRT-PCR. * *p* < 0.05, ** *p* < 0.01, *** *p* < 0.001, unpaired *t*-test.

To discover the pathways affected by miR-28-5p regulation we exploited the miRNA pull out assay [15] adequately revised [17]. This protocol allowed the capture and identification through next generation sequencing (NGS, RNAseq) of the RNAs interacting with miR-28-5p using a biotinylated version of miR-28-5p. The transcripts significantly enriched in the captured miR-28-5p transcriptome (see Material and Methods for more details and Supplementary Figure S2 for a flow chart of the overall study) were named the miR-28-5p targetome. The miR-28-5p targetome consisted of 191 putative targets, mostly of which (98%) (187 out of 191) were coding RNAs (Supplementary Table S1). To validate the reliability of targets identification, we ranked the 191 targets according to the RNAseq fold change enrichment and we randomly selected and measured the enrichment of eight of them by qRT-PCR. The results confirmed that all the selected targets were enriched (Figure 3A). We then evaluated the enrichment of miR-28-5p predicted targets in miR-28-5p targetome using three different miRNA-target prediction algorithms (TargetScan 7, PITA and RNA22). We found that the 61% (117 out of 191) of the targets were predicted by at least one algorithm (Figure 3B and Supplementary Table S2) supporting the validity of the method to identify miRNA targets.

We considered only the targets predicted by at least one prediction algorithm. To identify the pathways and the biological processes to which the selected targets belonged to, we first performed gene set enrichment analysis using the GSEA online tool, but we did not identify significantly (FDR ≤ 0.01) enriched pathways. Therefore, we shifted to a different bioinformatics approach, and we evaluated whether the selected miR-28-5p targets belonged to gene families using the Molecular Signature database (MSigDB) gene sets used by GSEA, which categorizes genes in gene families with annotated function. In this way, we discovered that the most enriched gene family was the “transcription factor” family (Figure 3C) that includes genes such as RNF41, ZNF25, ZNF22 (predicted by TargetScan 7), SREBF2, ZNF264 (predicted by RNA22) and EZH1 (predicted by Pita, TargetScan 7 and RNA22). We verified whether the six selected targets were enriched in miR-28-5p targetome by qRT-PCR and validated the enrichment of four out of six targets (Figure 3D).

### 3.3. miR-28-5p Directly Regulated SREBF2 Expression

**Figure 4:**
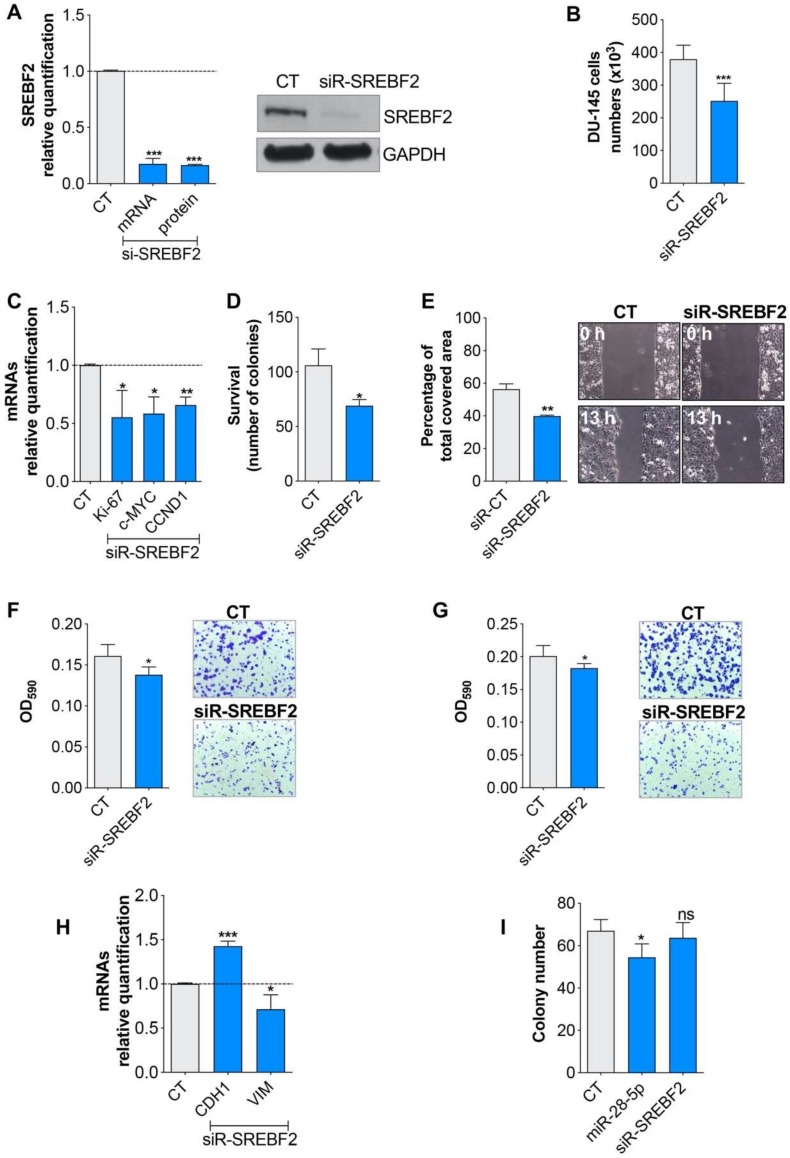
Evaluation of the SREBF2 role in DU-145 PCa cells. (**A**) SREBF2 inhibition by siR-SREBF2 transfection in DU-145 cells analyzed by qRT-PCR and western blot analysis. Evaluation of SREBF2 silencing effect on proliferation (**B**), survival (**D**) migration (**E**,**F**) and invasion (**G**) in DU-145 cells. Relative expression of proliferation (Ki-67, c-MYC and cyclin D1 (CCND1)) (**C**), epithelial (E-cadherin 1 (CDH1)) and mesenchymal markers (Vimentin (VIM)) (**H**) in miR-28-5p overexpressing versus normal DU-145 cells. (**I**) Number of colonies formed in soft agar in DU-145 cells after miR-28-5p overexpression or SREBF2 silencing. * *p* < 0.05, ** *p* < 0.01, *** *p* < 0.001, unpaired *t*-test. ns, not significant.

We focused on the transcription factor SREBF2 as it is a potential oncogenic transcription factor in prostate cancer [24]. To verify the role of SREBF2 in prostate cancer cells, we silenced SREBF2 in DU-145 cells and 48 h later we evaluated several cellular readouts. We found that the SREBF2 inhibition (Figure 4A) reduced cell proliferation (Figure 4B,C), survival (Figure 4D) as well as migration (Figure 4E,F) and invasion (Figure 4G), measured by both wound healing and trans-well assays. Even in this case the expression of the epithelial/mesenchymal markers was in accordance with the migration/invasion data (Figure 4H). On the contrary, SREBF2 silencing did not affect the anchorage-independent growth (Figure 4I). These results suggest that the inhibition of SREBF2 was able to affect the majority of the tumor processes affected by miR-28-5p re-expression.

**Figure 5:**
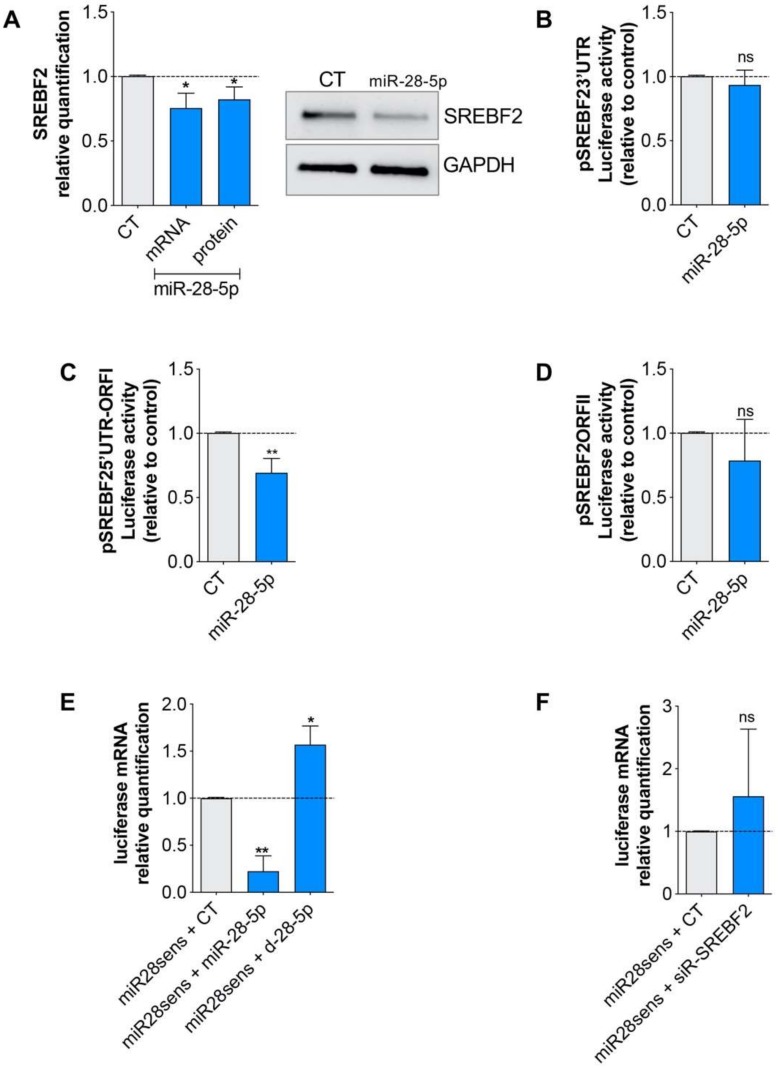
Analysis of miR-28-5p/SREBF2 interaction in PCa cells. (**A**) Quantification of SREBF2 mRNA and protein level in miR-28-5p versus CT transfected DU-145 cells. Relative luciferase activity after the cotransfection of pSREBF2_3′UTR_ (**B**), pSREBF2_5’UTR-ORFI_ (**C**), pSREBF2_ORFII_ (**D**) and either CT or miR-28-5p. (**E**) Relative luciferase mRNA level, analyzed with qRT-PCR, in DU-145 cotransfected with miR-28-5p sensor and miR-28-5p mimic or inhibitor versus miR-28-5p sensor/CT cotransfected cells. (**F**) Relative luciferase mRNA level, analyzed by qRT-PCR, in DU-145 cotransfected with miR-28-5p sensor/siR-SREBF2 versus miR-28-5p sensor/CT cotransfected cells. * *p* < 0.05, ** *p* < 0.01, *** *p* < 0.001, unpaired *t*-test. ns, not significant.

We then verified whether miR-28-5p regulated SREBF2. We transfected miR-28-5p in DU-145 cells and found that miR-28-5p overexpression determined a slight but significant decrease of SREBF2 expression both at the mRNA and protein levels (Figure 5A). The same results were obtained with the androgen-dependent PCa cell line LNCaP in which miR-28-5p overexpression, and SREBF2 inhibition determined a decrease of cells proliferation (Supplementary Figure S4A–C) and miR-28-5p negatively-regulated SREBF2 expression (Supplementary Figure S4D). To validate miR-28-5p interaction with SREBF2, we performed the luciferase reporter assay using a reporter vector encompassing SREBF2 3′UTR, and unexpectedly, we discovered that miR-28-5p did not regulate SREBF2 by interacting with the 3′UTR (Figure 5B). As it has already been demonstrated that miRNAs can regulate gene expression also by interacting with the ORF [25] or the 5′UTR [26] of their targets, we used RNAhybrid to verify whether the SREBF2 5′UTR-ORF contained putative miR-28-5p recognition sites, and we found three putative sites of interaction in the CDS region (Supplementary Table S3). It is of note that the majority of the binding sites were predicted also by RNA22 and PITA (Supplementary Table S3). We produced two luciferase reporter vectors, one encompassing the 5′UTR plus the first part of the ORF (pSREBF2_5’UTR-ORFI_, that includes one putative recognition site), and the other encompassing the second part of the ORF (pSREBF2_ORFII_, that includes two putative recognition sites) (Supplementary Table S3). We performed a luciferase reporter assay and found a significant reduction of luciferase activity after miR-28-5p overexpression only with pSREBF2_5’UTR-ORFI_ (Figure 5C,D). Overall, these data suggested that miR-28-5p repressed SREBF2 expression by interacting with the SREBF2 CDS region.

Finally, to evaluate whether the SREBF2/miR-28-5p interaction may also affect the availability of free miR-28-5p, we considered the possibility that SREBF2 might function as a sponge for miR-28-5p. For this purpose, we built a miR-28-5p sensor (miR28sens), consisting of a luciferase reporter vector in which two miR-28-5p perfect match sequences were cloned downstream of the luciferase gene. We first verified the capability of this sensor to detect miR-28-5p variations by co-transfecting the sensor with a miR-28-5p mimic or inhibitor, and then evaluating the luciferase mRNA variation by qRT-PCR: as expected the luciferase mRNA levels decreases or increases when the miR-28-5p was overexpressed or inhibited, respectively (Figure 5D). Then we tested SREBF2 sponge activity by co-transfecting the sensor with a SREBF2 inhibitor or control, but we did not detect any significant variation of the luciferase mRNA, suggesting that SREBF2 did not act as a sponge of miR-28-5p.

## 4. Discussion

Although in some tumor types an increased expression level of miR-28-5p has been reported [27,28,29], most of the papers regarding the role of this miRNA in cancer suggested a prevalent TS activity. We already demonstrated that miR-28-5p is involved in both tumor cell proliferation and survival by inhibiting cell proliferation and colony forming ability, and by inducing apoptosis and increasing the percentage of DU-145 cells in G1 phase [10]. In this paper we also evaluated whether this miRNA also plays a role in the capability of the tumor cells to migrate and invade, finding that miR-28-5p re-expression determined a reduction of the PCa cells capability to grow unattached to a matrix and also to migrate and invade. These results were in line with the fact that miR-28-5p expression tends to decrease in tumor tissues and is more severely diminished in metastatic tissues. These data indicated that miR-28-5p exerts its TS activity by affecting almost all the aspects of tumor biology, including the cellular processes fundamental for tumor development and progression, although this is not true for all cancer types [6]. In addition, we discovered that miR-28-5p expression was also associated with two fundamental clinicopathological characteristics of prostate cancer progression (pathological T stage and the Gleason score), as well as with biochemical relapse. These data reinforce the TS activity of miR-28-5p in PCa and, as already demonstrated in gastric and colorectal cancer [6,30], suggesting a possible role of this miRNA as a prognostic biomarker. Another point is that we demonstrated in vitro that miR-28-5p re-expression could reduce processes linked to tumor growth. In line with our observations are the data obtained by Bartolomé-Izquierdo et al. [31] which demonstrated that the miR-28-5p re-expression blocked tumor growth in a Burkitt lymphoma murine model, opening the way to a possible therapeutic use of miR-28-5p (replacement therapy). All these aspects justified the interest to identify the miR-28-5p targetome in PCa.

The exploitation of the miRNA pull-out assay technique optimized with NGS technology allowed us to identify the miR-28-5p targetome in PCa that consists of 191 targets. Considering the miR-26a-5p targetome of DU-145 cells obtained in our previous work [17] we observed that miR-28-5p exerts its TS activity by interacting with a relative lower number of targets respect to miR-26a-5p (1423 targets). The fact that miR-28-5p re-expression regulates fewer genes than miR-26a-5p was suggested also by the proteome analysis, performed using mass spectrometry, after miRNAs re-expression (preliminary data). The reliability of the method in capturing miR-28-5p targets was demonstrated by the fact that 61% (117 targets) of miR-28-5p targets presented at least one predicted binding site for miR-28-5p. This evaluation was done using three different prediction algorithms that consider canonical and non-canonical miRNA/target interactions within the 3′UTR of the targets. We focused on targets with at least one miR-28-5p predicted binding site and, to catch the targets whose function could be relevant for miR-28-5p activity, we tested whether some pathways or biological processes were enriched by the targets. Unfortunately, the analysis did not show pathways significantly enriched by miR-28-5p targets possibly due to the low number of targets available for the analysis. However, we found that that some targets belonged to gene families with annotated functions, and in particular, the largest family was the “transcription factor family”. Given that a transcription factor regulates in turn several genes, we focused on this family, and in particular on SREBF2 (sterol regulatory element-binding protein 2), an essential regulator of genes associated with cholesterol biosynthesis [32]. Aberrant SREBF2 expression and activity has been associated with PCa progression [24,33,34], probably due to the increasing need of proliferating cells for the key component of cell membranes such as cholesterol and other lipids. Indeed, SREBP metabolic pathway impairment is under evaluation as an effective anti-tumor therapy [35]. In accordance with these observations we demonstrated that the inhibition of SREBF2 in DU-145 cells induced an anti-tumor activity also in this case by affecting various tumor characteristics such as cell proliferation, survival and to a lesser extent, invasion and migration. It is of note that SREBF2 inhibition did not affect the anchorage-dependent growth of DU-145 cells. Since the silencing of SREBF2 was not able to recapitulate all the effects of miR-28-5p re-expression, these results suggested that SREBF2 is not the only mediators of miR-28-5p TS activity. In addition, Chen et al. demonstrated that the reactivation of the MAPK pathway determined an up-regulation of SREBP proteins promoting metastatic PCa [34]. Given that MAPK1 has been shown to be a miR-28-5p target in myeloproliferative neoplasm [36], it is possible that miR-28-5p regulates SREBF2 both directly and indirectly through MAPK. It is of note that MAPK1 is included in the miR-28-5p targetome (Supplementary Tables S1 and S2).

Finally, during the validation experiments we demonstrated that miR-28-5p re-expression determines a slight but significative reduction of SREBF2 expression at both the protein and mRNA levels, and that miR-28-5p regulates SREBF2 expression through the interaction with SREBF2 ORF. Although miRNAs binding of the ORFs are generally considered less effective [37,38], there are several lines of evidence that confirmed that sites in both 5′UTRs and ORFs can mediate target repression [25,39,40,41]. Moreover, the fact that Helwak et al. [14] using the CLASH technique on HEK293 cells found the interaction between miR-28-5p and the first part of the ORF of SREBF2 further reinforces our findings.

In conclusion, we demonstrated that miR-28-5p had a TS activity on PCa cells by affecting several fundamental processes of tumor development and progression. We showed that the miRNA pull-out assay represents a valid tool to isolate the targetome of a specific miRNA. We demonstrated that SREBF2 is one of the mediators of the miR-28-5p TS activity, given that its inhibition determined a decrease of tumor cell survival and proliferation and to a lesser extent invasion and migration. Our findings reinforce the concept that the identification of the targetome of a specific miRNA with anti-tumor activity allows the identification of new genes or pathways with a role in cancer development and hence possible therapeutic targets.

## Supplementary Materials

The following are available online at https://www.mdpi.com/2073-4409/9/2/354/s1, Table S1: Transcripts of miR-28-5p targetome identified by NGS, Table S2: miR-28-5p targetome predicted targets, Table S3: miR-28-5p binding sites predicted by RNAhybrid in SREBF2 transcript. The binding sites predicted also by RNA22 and PITA are indicated, Figure S1: Schematic representation of synthetic oligos used for miRNA pull out assay. Thiouridines (red) and mutations inserted in the antisense strands (blue) are indicated, Figure S2: Schematic representation of overall study consisting in the miR-28-5p targets capture (through miRNA pull-out assay) and identification (through NGS and data analysis) to obtain the miR-28-5p targetome, Figure S3: (A) Analysis of the LPP expression in prostate cancer cell lines with respect to normal cells. (B) Comparison of LPP (*x* axis) and miR-28-5p (*y* axis) expression levels in MSKCC study’s patients. Pearson correlation and *p*-value test are indicated. (C) Kaplan-Maier curves and results of the recurrence-free survival analysis of MSKCC patients using LPP expression level as discriminant for the two groups. Long-rank *p*-value test is shown, Figure S4: (A,B) Proliferation after SREBF2 silencing of LNCaP cells. (C) Relative quantification of proliferations markers (Ki-67 and c-MYC) after miR-28-5p overexpression (miR-28-5p) or SREBF2 silencing (siR-SREBF2) in LNCaP cells. (D) Quantification of SREBF2 mRNA level in miR-28-5p versus CT transfected LNCaP cells.
