# Supplementary figures and images for: The miR-28-5p Targetome Discovery Identified SREBF2 as One of the Mediators of the miR-28-5p Tumor Suppressor Activity in Prostate Cancer Cells
Source: Cells. 2020 Feb 3;9(2):354. doi: 10.3390/cells9020354 (PMC7072282; doi:10.3390/cells9020354)

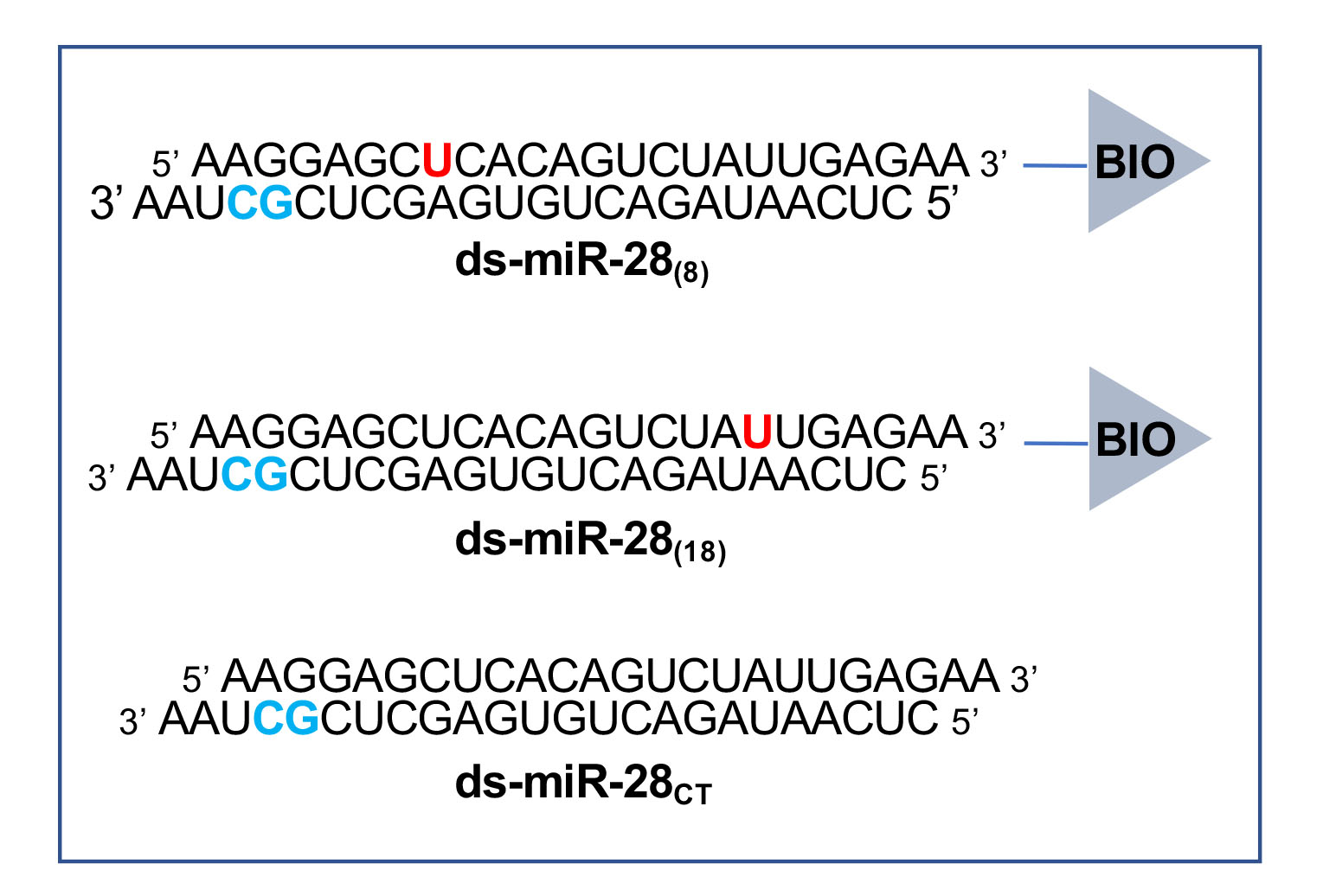

Supplement: Supplementary file 1 [file cells-09-00354-s001.zip › Supplementary files/Supplementary Figure S1.jpg]

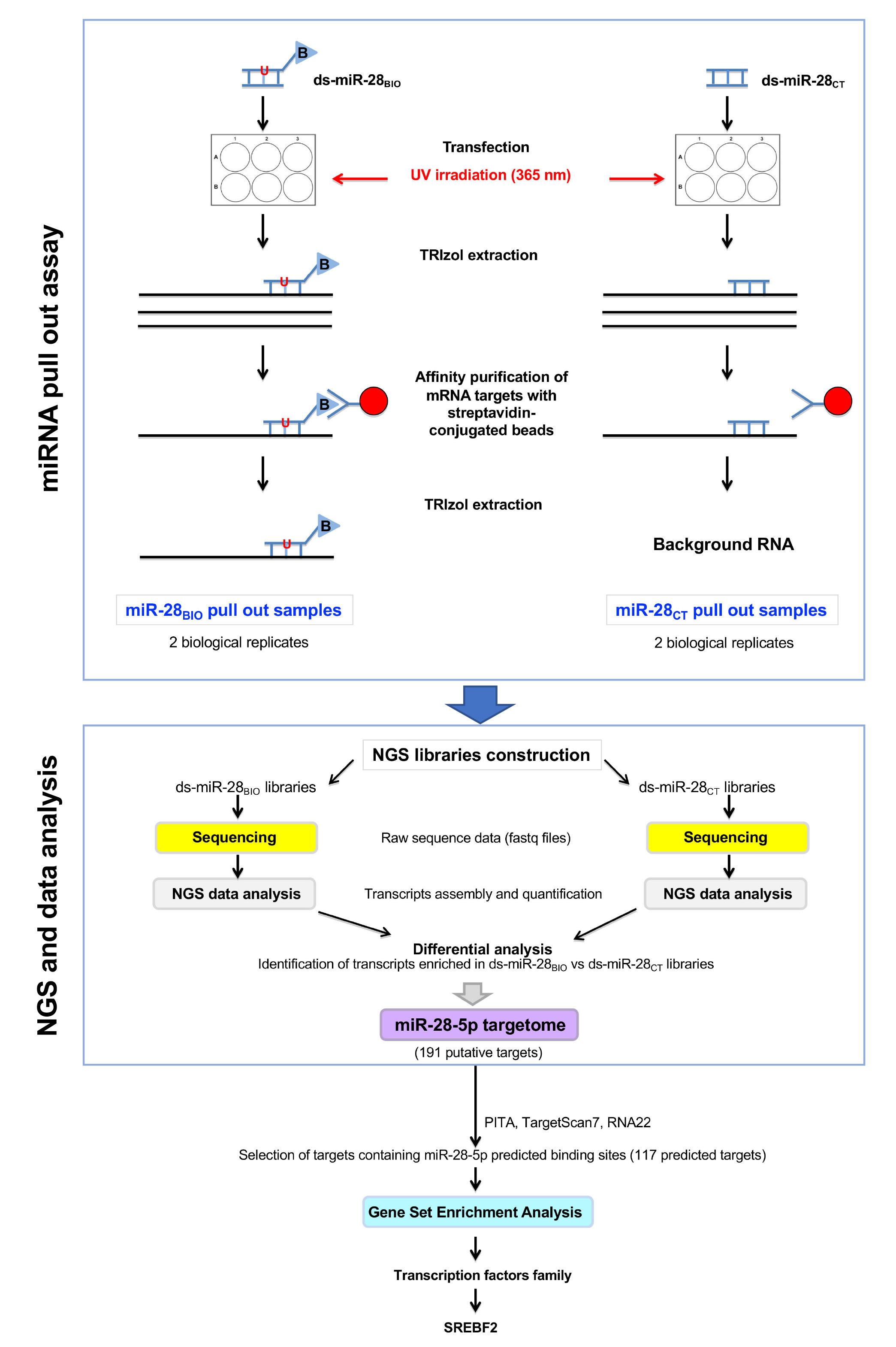

Supplement: Supplementary file 1 [file cells-09-00354-s001.zip › Supplementary files/Supplementary Figure S2.jpg]

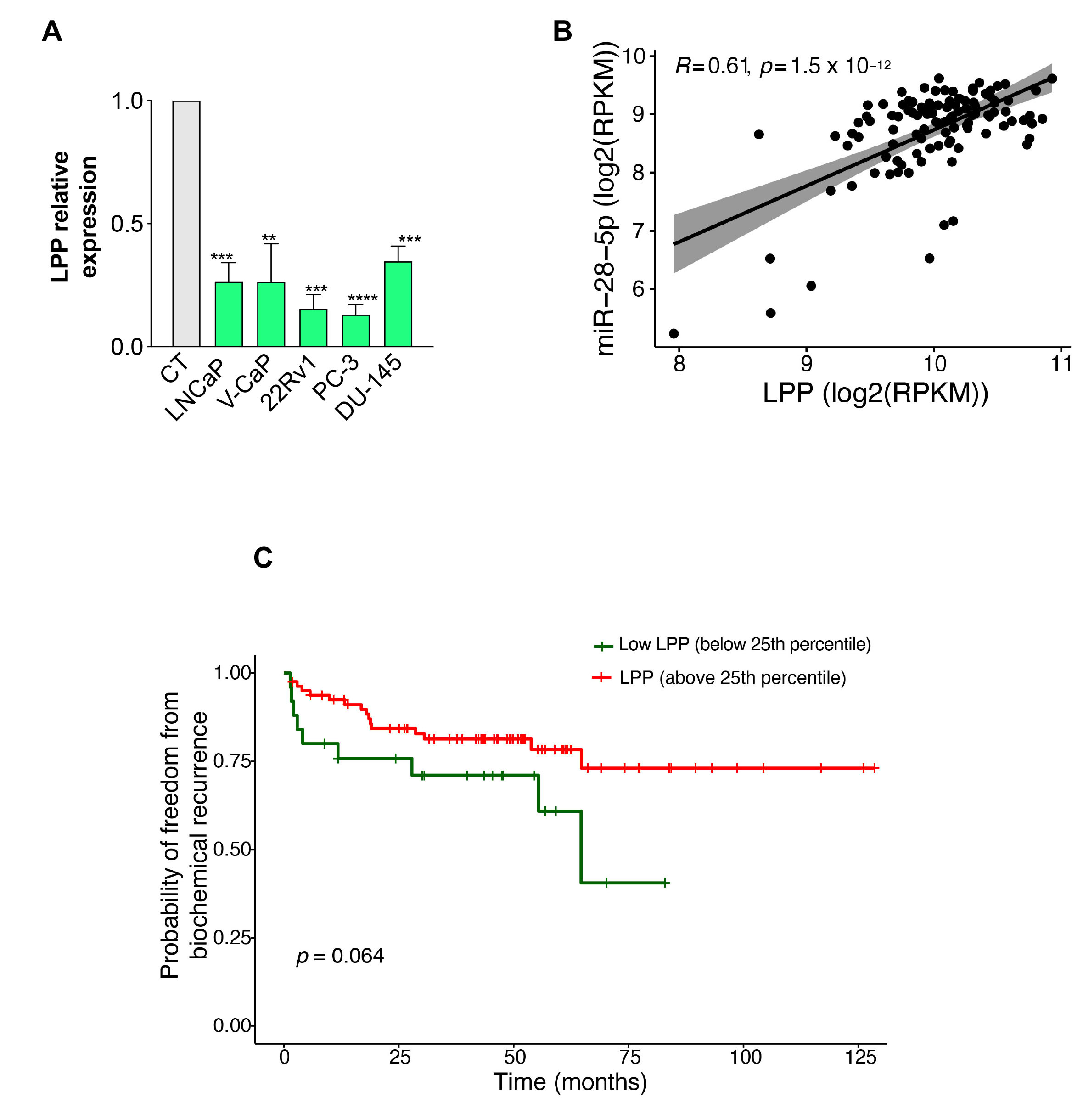

Supplement: Supplementary file 1 [file cells-09-00354-s001.zip › Supplementary files/Supplementary Figure S3.jpg]

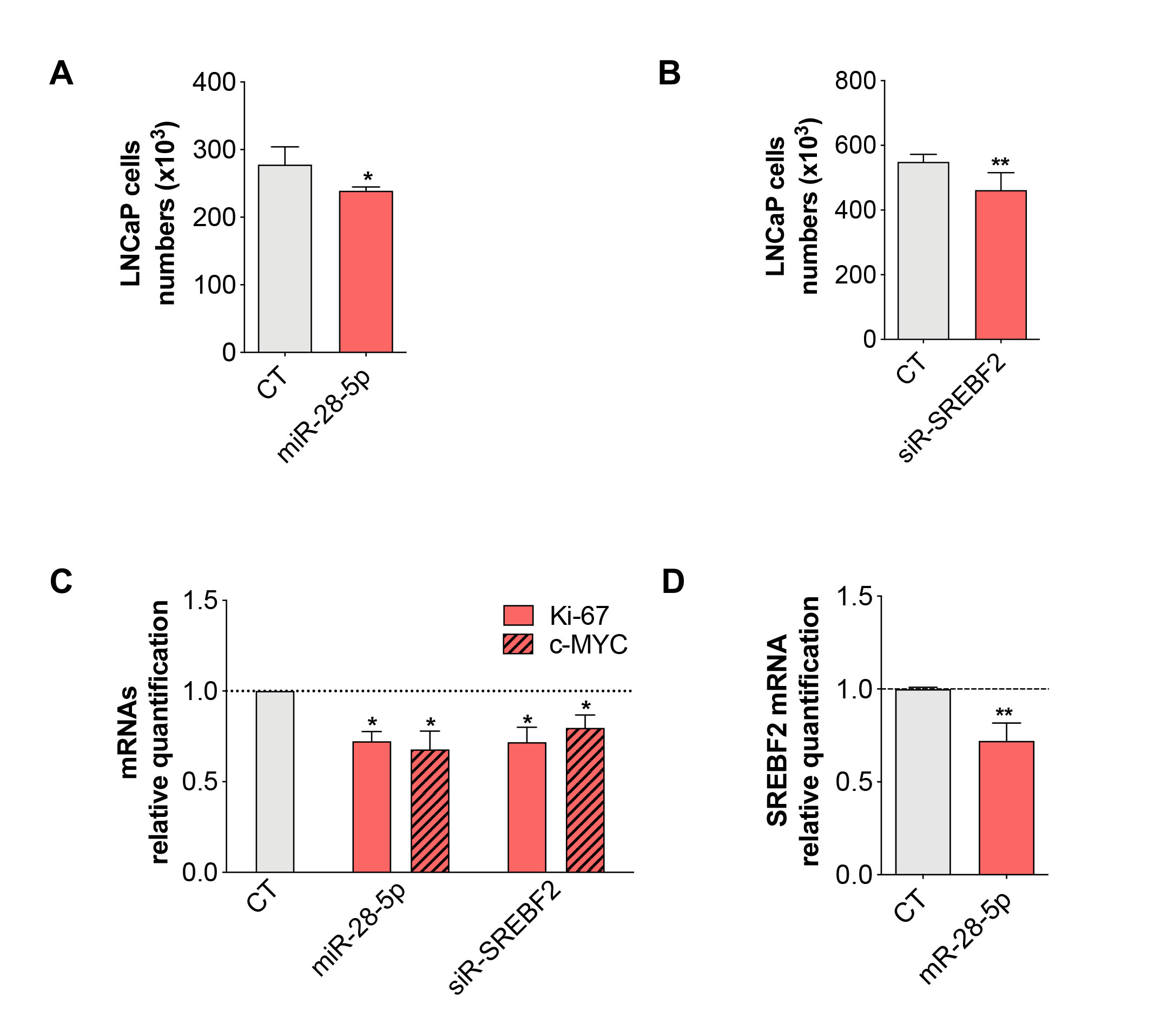

Supplement: Supplementary file 1 [file cells-09-00354-s001.zip › Supplementary files/Supplementary Figure S4.jpg]
